# Perioperative hemoglobin area under the curve is an independent predictor of renal failure after cardiac surgery. Results from a Spanish multicenter retrospective cohort study

**DOI:** 10.1371/journal.pone.0172021

**Published:** 2017-02-22

**Authors:** Paula Duque-Sosa, Diego Martínez-Urbistondo, Gemma Echarri, Raquel Callejas, María Josefa Iribarren, Gregorio Rábago, Pablo Monedero, Spanish group of renal dysfunction in cardiac surgery (GEDRCC-2)

**Affiliations:** 1 Department of Anesthesia and Critical Care, Clínica Universidad de Navarra, Pamplona, Navarra, Spain; 2 Department of Internal Medicine, Division of Intermediate Care and Hospitalists Unit, Clínica Universidad de Navarra, Pamplona, Navarra Spain; 3 Department of Cardiovascular Surgery, Clínica Universidad de Navarra, Pamplona, Navarra, Spain; University of Sao Paulo Medical School, BRAZIL

## Abstract

Perioperative anemia is an important risk factor for cardiac surgery-associated acute kidney injury (CSA-AKI). Nonetheless, the severity of the anemia and the time in the perioperative period in which the hemoglobin level should be considered as a risk factor is conflicting. The present study introduces the concept of perioperative hemoglobin area under the curve (pHb-AUC) as a surrogate marker of the evolution of perioperative hemoglobin concentration. Through a retrospective analysis of prospectively collected data, we assessed this new variable as a risk factor for the development of acute kidney injury after cardiac surgery in 966 adult patients who underwent cardiac surgery with cardiopulmonary bypass, at twenty-three academic hospitals in Spain. Exclusion criteria were patients on renal replacement therapy, who needed a reoperation because of bleeding and/or with missing perioperative hemoglobin or creatinine values. Using a multivariate regression analysis, we found that a pHb-AUC <19 g/dL was an independent risk factor for CSA-AKI even after adjustment for intraoperative red blood cell transfusion (OR 1.41, p <0.05). It was also associated with mortality (OR 2.48, p <0.01) and prolonged hospital length of stay (4.67 ± 0.99 days, p <0.001)

## Introduction

Acute kidney injury (AKI) is a common and serious complication of cardiac surgery (CS). It occurs with various degrees of severity in up to 30% of patients, with about 1–2% of these requiring dialysis [1–3]. The development of AKI, even when mild, is independently associated with an increase in morbidity and mortality [4–6]. Moreover, there are currently no clinically proven therapies that can prevent or treat cardiac surgery-associated acute kidney injury (CSA-AKI) [1,7,8]. For this reason, targeting modifiable risk factors is mandatory to minimize the incidence of AKI in these patients [9].

Previous studies have identified important risk factors for CSA-AKI such as female gender, history of chronic obstructive pulmonary disease (COPD), diabetes mellitus, peripheral vascular disease, pre-existing renal insufficiency, congestive heart failure, left ventricular ejection fraction (LVEF) < 35%, emergency surgery, cardiogenic shock requiring insertion of intra-aortic balloon pulsation (IABP), left main coronary artery disease, length of cardiopulmonary bypass (CPB), duration of aortic cross-clamping and valve surgery [3,10–13]. While most are not modifiable, anemia, perioperative red blood cell (RBC) transfusion and surgical-re-explorations have been identified as potentially modifiable risk factors [9,14,15].

Nonetheless, the severity of the anemia and the time in the perioperative period in which the hemoglobin (Hb) level should be considered as a risk factor is conflicting. For this reason, we developed a new marker of perioperative anemia defined as the perioperative hemoglobin area under the curve (pHb-AUC). It could be used as a surrogate marker of the evolution of the perioperative hemoglobin concentration in cardiac surgery and maybe a better indicator of the “anemic status”.

The purpose of this study is to assess this new variable, as a risk factor for the development of CSA-AKI.

## Patients and methods

Twenty-three academic hospitals in Spain participated in this observational multicenter study that is based on a retrospective analysis of prospectively collected data. The cohort includes 966 consecutive patients aged >18 years, who underwent CS with CPB, from October 2012 to March 2013. Investigators at each hospital collected detailed perioperative data using standardized case reports forms. The authors entered the records into a computerized database. Demographics, laboratory tests, type of surgery, past medical history, comorbidities, previous CS, medication (ACE-inhibitors, ARBs, NSAIDs, anticoagulants, statins), use of contrast media in the previous 72 hours, the time between catheterization and surgery, requirement of IABP, use of intravenous inotropes, intraoperative or postoperative use of blood products, length of intensive care unit (ICU) and hospital stay, were included in the database. We did not use a specified protocol to guide transfusion therapy across all participating sites, due to the nature of this observational and non-interventional study. Cases involving heart transplantation, repair of congenital abnormalities and ventricular assist device placement were excluded because these procedures were not performed at all participating hospitals. Exclusion criteria included patients already on renal replacement therapy (RRT), who needed a reoperation because of bleeding and/or with missing perioperative hemoglobin or creatinine values. These exclusion criteria were applied by each center before the inclusion of patients in the data base.

### Ethical standards

The study protocol was approved on March 2012 by the Institutional Review Board (IRB) from the health department of the government of Navarra (EO 3/2012). Each participating center was responsible for informing its local ethics committee of the approval at regional level, which authorizes the study to be carried out at the national level. Although it is an observational study, written informed consent was obtained from all patients because the data were collected prospectively. The study was performed in accordance with the ethical of the 1964 Declaration of Helsinki and its later amendments.

### Primary independent variables

The independent variables were preoperative Hb concentration, the lowest intraoperative Hb, the first postoperative Hb and RBC transfusion on the day of surgery.

The preoperative Hb concentration was defined as the Hb value of the day before surgery (Hb1). Because it is an observational multicenter study, there is no specific protocol for time samples through the intraoperative period. Nonetheless, the different centers of cardiac surgery in Spain have standardized the frequency and timing for Hb determinations. In current practice, Hb determinations were just before CPB, then two samples during CPB and after CPB. The lowest intraoperative Hb value is shown as Hb2. The first postoperative Hb was the sample taken at intensive care unit admission (Hb3).

### Dependent variable and AKI definition

The primary dependent variable was the incidence of postoperative AKI defined according to the Kidney Disease: Improving Global Outcomes (KDIGO) [16].

Stage 1: increase in serum creatinine of ≥0.3 mg/dl within 48 hours or increase in serum creatinine 1.5 to 1.9 times from baseline which is known or presumed to have occurred within the first week after surgery.
Stage 2: increase in serum creatinine to 2 to 2.9 times from baseline which is known or presumed to have occurred within the first week after surgery.
Stage 3: increase in serum creatinine to 3 times from baseline or increase in serum creatinine ≥4 mg/dl or need for RRT which is known or presumed to have occurred within the first week after surgery.

For calculation purposes, creatinine values before and after surgery (daily for 7 days) were routinely measured in all patients. Baseline creatinine was considered as the most recent available preoperative creatinine value. AKI was considered as a dichotomic variable (present or absent) and as an ordinal variable in the different 3 stages.

### Hemoglobin AUC calculation

**Fig 1:**
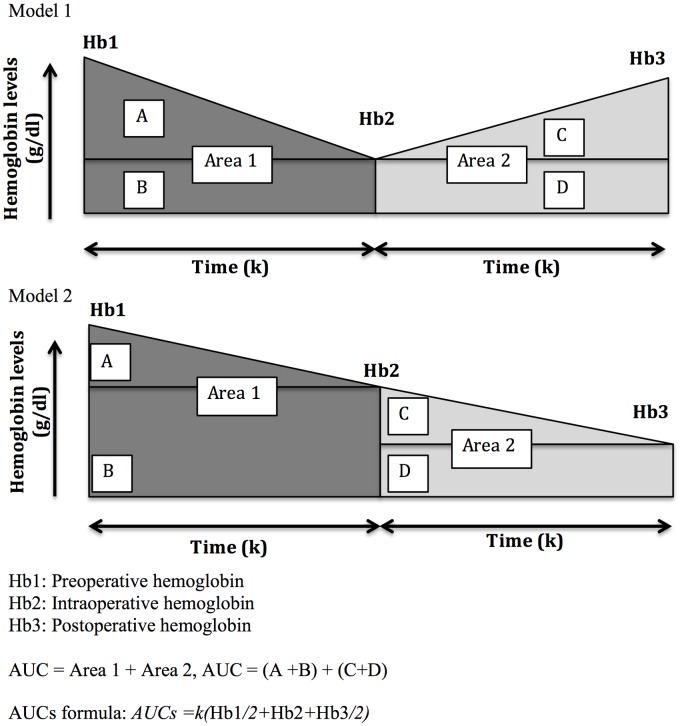
Calculation of perioperative hemoglobin area under the curve (AUC). *Hb1*: *Preoperative hemoglobin* *Hb2*: *Intraoperative hemoglobin* *Hb3*: *Postoperative hemoglobin* *AUC = Area 1 + Area 2*, *AUC = (A+B) + (C+D)* *AUCs formula*: *AUCs = k (Hb1/2+Hb2+Hb3/2)*

To assess the influence of hemoglobin evolution on the development of AKI, a model of hemoglobin area under the curve was developed. This model is a simplified version of AUC standard calculation. We used this concept because it could be a surrogate marker of the “perioperative hemoglobin evolution” in cardiac surgery and may possibly be a better indicator of the “anemic status” than isolated values of hemoglobin in the perioperative period. The perioperative hemoglobin area under the curve was calculated as the concentration between preoperative hemoglobin levels (Hb1) and the minimum intraoperative hemoglobin (Hb2), plus the minimum Hb2 and the postoperative hemoglobin (Hb3). For calculation purposes, the period of time between Hb1, Hb2 and Hb3 (*k*) was considered equal and constant (Fig 1) in all patients and in both periods. The (*k*) was considered as a constant, because the purpose of the study was to obtain a practical assessment of the perioperative hemoglobin concentration and the “real time” between the different periods of Hb determinations would have been difficult, cumbersome and with limited value for future use.

The mathematical algorithm to calculate simplified AUC (AUCs) is as follows:

AUC = Area 1 + Area 2

AUC = (A+B) + (C+D)

If Hb2 < Hb3 apply model 1

AUCs = (kHb2) + [k(Hb1-Hb2)/2] + k(Hb2) + [k(Hb3-Hb2)/2]

AUCs = k (Hb1+Hb2)/2 + k (Hb2+Hb3)/2

AUCs = k (Hb1/2+Hb2+Hb3/2)

If Hb2 >Hb3 apply model 2

AUCs = (kHb2) + [k(Hb1-Hb2)/2] + k (Hb3) + [k (Hb2-Hb3)/2]

AUCs = k (Hb1+Hb2)/2 + k (Hb2+Hb3)/2

AUCs = k (Hb1/2+Hb2+Hb3/2)

Both models allow an easy calculation of AUCs with the following formula:

AUCs = k (Hb1/2+Hb2+Hb3/2)

### Statistical analysis

Continuous variables were reported as mean and standard deviation. These variables were analysed by t-Student’s test, and U-Mann Whitney tests. Categorical variables were summarized as absolute frequencies and percentages, and analysed by chi-square test. Fisher´s exact test was used for comparison of dichotomic variables in which the estimated sample size was small. Sensitivity analysis was performed with area under receiver operation characteristic (AUROC) curve. ROC curve analysis was used to compare the performance of Hb1, Hb2 and pHb-AUC in predicting CSA-AKI. Multivariate analysis was performed using binary logistic regression and linear regression. Variables were introduced in the model when they were statistically significant in the prediction of the outcome (p <0.05) in the univariate analysis. When collinearity was present between measurements, the variables with a higher prevalence and those related to mortality prediction were selected. Statistical analyses was performed, using SPSS for Windows, version 20.0 (SPSS Inc, Chicago,IL).

## Results

During the study period, 975 patients underwent cardiac surgical procedures with CPB. A total of 9 patients met one or more of the exclusion criteria and were excluded from analyses, leaving 966 consecutive patients in the study.

Patient characteristics are described in Table 1. Our cohort consisted mainly of men (63.3%) undergoing valve surgery alone (51.5%) or combined with CABG (12.7%). The main comorbidities were congestive heart failure (24%), pre-existing renal dysfunction (basal creatinine >1.2 mg/dL) (11%), chronic obstructive pulmonary disease (13.3%), diabetes mellitus (28.2%), peripheral vascular disease (12.3%) and neurological dysfunction (disease severely affecting ambulation or day to day functioning) (5.4%). The estimated mortality surgical risk, based on European System for Cardiac Operative Risk (EuroSCORE) [17] was 14.1 ± 3.1%. A total of 66 (6.8%) had emergency surgery and 41 (4.2%) had previous CS.

**Table 1:** Patient characteristics.

Population characteristics	n = 966
Age	66.48 (12.35)
BMI	27.74 (4.49)
Caucasian race, n (%)	957 (99.1)
Male, n (%)	611 (63.3)
**Comorbidities**	
Chronic renal failure, n (%)	106 (11.0)
COPD, n (%)	128 (13.3)
Diabetes mellitus, n (%)	272 (28.2)
Neurologic dysfunction, n (%)	52 (5.4)
Peripheral vascular disease, n (%)	119 (12.3)
**Cardiac characteristics**	
Acute myocardial infarction, n (%)	67 (6.9)
Congestive heart failure, n (%)	232 (24.0)
Emergency surgery, n (%)	66 (6.8)
Endocarditis, n (%)	45 (4.7)
Preoperative ejection fraction	56.51 (12.1)
Previous cardiac surgery, n (%)	41 (4.2)
Unstable angina, n (%)	108 (11.2)
**EuroSCORE**	
EuroSCORE	14.1 (3.14)
**Type of surgery**	
CABG, n (%)	346 (35.8)
Combined, n (%)	123 (12.7)
Valvular, n (%)	497 (51.5)
**Hemoglobin levels**	
Hemoglobin (g/dl)	13.31 (1.86)
Preoperative anemia*, n (%)	300 (31.1)

Values are expressed as mean (standard deviation), unless otherwise stated.

BMI: body mass index; CABG: coronary artery bypass grafting COPD: chronic obstructive pulmonary disease; EuroSCORE: European System for Cardiac Operative Risk.

*Anemia was defined as <13 g/dl in men and <12 g/dl in women.

Perioperative variables and main outcomes are described in Table 2. Total observed in-hospital mortality was 4.7% (45/966), ICU and total length of hospital stay were 5.0 ± 8.4 and 15.1 ± 15.4 days, respectively.

**Table 2:** Perioperative variables and main outcomes.

Intraoperative variables	n = 966
Aortic cross clamp time (minutes)	79.3 (48.3)
Cardiopulmonary bypass time (minutes)	109.3 (52.4)
Intraoperative diuretic use, n (%)	262 (27.1)
Intraoperative anemia*, n (%)	386 (40.0)
Intraoperative RBC transfusion, n (%)	509 (52.7)
Intraoperative vasopressor use, n (%)	457 (47.3)
Minimum intraoperative hemoglobin	8.4 (1.7)
**Postoperative variables**	
Postoperative minimum hemoglobin	9.5 (1.7)
Postoperative transfusion requirement, n (%)	408 (42.2)
**pHb-AUC**	19.8 (2.7)
**Outcomes**	
AKI	360 (37.3%)
KDIGO 1 (% of AKI)	212 (58.9%)
KDIGO 2 (% of AKI)	75 (20.8%)
KDIGO 3 (% of AKI)	73 (20.3%)
Hospital length of stay	15.1 (15.4)
ICU length of stay	5.0 (8.4)
Overall hospital mortality, n (%)	45 (4.7)
Renal replacement therapy, n (%)	40 (4.1)

Values are expressed as mean (standard deviation), unless otherwise stated.

AKI: acute kidney injury; ICU: intensive care unit; KDIGO: Kidney Disease: Improving Global Outcomes; pHb-AUC: perioperative hemoglobin area under the curve; RBC: red blood cells

*Anemia was defined as Hb < 8 g/dl

### Acute Kidney Injury (AKI)

A total of 360 patients (37.3%) developed AKI and 40 (4.1%) required RRT (Table 2).

In the univariate analyses, AKI was significantly associated with preoperative factors such as advanced age, male gender, body mass index (BMI), history of CHF with reduced LVEF, diabetes mellitus, chronic renal failure, previous cardiac surgery, emergency surgery and high EuroSCORE (Table 3). Predictive intraoperative factors were: aortic cross clamp time, length of CPB and intraoperative use of vasopressors (Table 3). In-hospital mortality of patients with CSA-AKI was 11.1% (40/360) in contrast with 0.8% (5/606) of the population without AKI (p <0.001). ICU and total length of hospital stay were also significantly higher in patients with CSA-AKI (7.1 vs 3.8 and 18.5 vs. 13.1 days respectively, p <0.001). Potential confounders such as previous use of ACE-inhibitors, ARBs, NSAIDs, and contrast media (less than 10% of the cohort), were not significantly related with AKI development. Other nephrotoxic drugs, such as aminoglycosides and amphotericin, were not included in data collection due to the infrequent use in this population.

**Table 3:** Univariate analysis of risk factors for AKI after cardiac surgery.

Variable	No AKI (n: 606)	AKI (n: 360)	p
**Demographics**			
Age*	64.5 (12.9)	69.8 (10.4)	<0.01
Preoperative Hb (Hb1)*	13.6 (1.8)	12.7 (1.9)	<0.01
BMI	27.4 (4.4)	28.3 (4.5)	<0.01
Male, n (%)	365 (60)	246 (68)	<0.01
**Comorbidities**			
Chronic renal failure*, n (%)	35 (5.7)	71 (19.7)	<0.01
Diabetes mellitus*, n (%)	154 (25)	118 (33)	<0.01
Preoperative anemia, n (%)	135 (22)	165 (45)	<0.01
**Cardiologic variables**			
Emergency surgery, n (%)	33 (5.4)	33 (9.2)	<0.05
Heart failure, n (%)	112 (18.4)	120 (33.3)	<0.05
Previous cardiac surgery, n (%)	13 (2.1)	28 (7.7)	<0.01
EuroSCORE*			
EuroSCORE*	13.5 (3.1)	14.9 (3.0)	<0.01
**Perioperative variables**			
Aortic cross clamp time	72.5 (33.1)	90.8 (65.0)	<0.01
CPB time*	99.6 (40.0)	125.4 (65.3)	<0.01
pHb-AUC*	20.2 (2.7)	19.0 (2.6)	<0.01
pHb-AUC <19 g/dl*, n (%)	211 (34.8)	190 (52.7)	<0.01
IO anemia, n (%)	215 (35.4)	171 (47.5)	<0.05
IO minimum hb (Hb2)*	8.6 (1.7)	8 (1.6)	<0.01
IO RBC transfusion*, n (%)	279 (46)	230 (63.8)	<0.01
IO vasopressors, n (%)	295 (48.7)	214 (59.4)	<0.01
**Hospital mortality, n (%)**	5 (0.8)	40 (11.1)	<0.01

Values are expressed as mean (standard deviation), unless otherwise stated.

AKI: acute kidney injury; BMI: body mass index; CPB: cardiopulmonary bypass; EuroSCORE: European System for Cardiac Operative Risk; pHb-AUC: perioperative hemoglobin area under the curve; IO: intraoperative; RBC: red blood cells.

*These variables also showed statistically significant results in predicting hospital mortality.

### Anemia and transfusion as risk factors for AKI

Anemia was defined as preoperative hemoglobin concentration of less than 13 g/dL for men and less than 12 g/dL for women, which is the threshold of the World Health Organization sex based definition [18]. The mean preoperative hemoglobin concentration was 13.3 ± 1.86 g/dL. Preoperative anemia was found in 31.1% (300/966) of our patients. Preoperative anemia was present in 45.8% (165/360) of patients with AKI and it was found to be an independent risk factor for CSA-AKI (OR 2.58, 95% CI: 1.92–3.45, p <0.01). Characteristics of our patients with CSA-AKI and their risk factors are shown in Table 3 (univariate analysis of risk factors for AKI) and Table 4 (individual logistic regression analysis for AKI including hemoglobin, anemia and RB transfusion).

**Table 4:** Anemia and hemoglobin AUC as risk factors for AKI in cardiac surgery*.

**Variable**	**OR**	**95% CI**	**p value**
Preoperative hemoglobin (Hb1)	0.80	0.74–0.87	<0.05
Preoperative anemia	2.58	1.92–3.45	<0.05
IO minimum hemoglobin (Hb2)	0.84	0.77–0.92	<0.05
IO anemia	1.32	1.00–1.75	0.05
pHb-AUC	0.85	0.80–0.91	<0.05
pHb-AUC <19 g/dl	1.75	1.30–2.35	<0.01

*Values were adjusted by transfusion

AKI: acute kidney injury; CI: confidence interval; IO: intraoperative; pHb-AUC: perioperative hemoglobin area under the curve; OR: odds ratio

Intraoperative anemia (IOA) was defined as an intraoperative hemoglobin ≤8 g/dL during CPB, as in previous studies [14,19]. The mean minimum intraoperative hemoglobin was 8.4 ± 1.7 g/dL. Intraoperative anemia was present in 47.5% (171/360) of patients with AKI and it was associated with an increased risk of CSA-AKI (OR 1.32, 95% CI: 1.00–1.75, p = 0.05).

RBC transfusions on the day of surgery were administered in 52.7% (509/966) of our whole cohort and the transfusion rate was 1.7 times higher in subjects with preoperative anemia (74% vs 43.1%, p <0.001). There were no RBC transfusions the day before surgery. Blood transfusion rate was higher, 88.5% (230/360), in patients who developed AKI, and it was associated with an increased risk of AKI (OR 2.10, 95% CI: 1.59–2.71, p <0.01).

### Perioperative hemoglobin area under the curve (pHb-AUC)

The mean pHb-AUC of our whole cohort was 19.8 ± 2.7 g/dL. The mean pHb-AUC was significantly lower in patients with AKI: 19.0 ± 2.6 compared with 20.2 ± 2.7 g/dL in patients without AKI (p <0.01). After the performance of the sensitivity analysis, a pHb-AUC of 19 g/dL was defined as the cut-off value for AKI risk discrimination. The sensitivity and specificity of this cut-off value were 0.53 and 0.61 respectively. Preoperative anemia, which is a well acknowledged parameter in CSA-AKI has a sensitivity of 0.55 and a specificity of 0.65 when analyzed alone.

Therefore, an AUC under 19 g/dL was significantly associated with AKI development (52.7% vs 34.8%, p <0.01) even adjusted by RBC transfusion (OR 1.75, 95% CI: 1.30–2.35, p <0.01) (Tables 3 and 4). ROC curve analysis was used to compare the performance of preoperative hemoglobin (Hb1) (AUROC: 0.63; CI 95% 0.59–0.66), intraoperative hemoglobin (Hb2) (AUROC: 0.61; CI 95% 0.57–0.64) and pHb-AUC (AUROC: 0.64; CI 95% 0.60–0.67) in predicting CSA-AKI. The pHB-AUC was slightly superior than the isolated values of hemoglobin. Additionally, a pHb-AUC < 19 g/dL was associated with mortality risk (OR 2.48, 95% CI: 1.31–4.76, p <0.01) and prolonged hospital length of stay (4.67 ± 0.99 more days, p <0.001).

### Multivariate analysis of risk factors for AKI

The logistic regression model, odds ratios, the 95% CI for the odds ratios, and p values, are shown in Table 5. In this final model, the advanced age, body mass index (BMI), history of CHF with reduced LVEF, pre-existing renal dysfunction, length of CPB, RBC transfusions on the day of surgery and pHb-AUC <19 g/dL, were significantly associated with CSA-AKI and AKIN stage (p <0.05). Preoperative anemia and intraoperative anemia were excluded from the final model in order to avoid collinearity with the pHb-AUC, although both of them were associated with AKI. The pHb-AUC <19 g/dL was the only hemoglobin measurement independent from RBC transfusion and related to CSA-AKI in the multivariate analysis.

**Table 5:** Multivariate regression logistic analysis of perioperative risk factors for AKI in cardiac surgery.

Variable	OR	95% CI	p value
**Preoperative**			
• Age (per year)	1.03	1.02–1.05	<0.01
• BMI (Kg/m^2^)	1.06	1.03–1.10	<0.01
• Chronic heart failure	1.69	1.21–2.35	<0.01
• Chronic renal insufficiency	3.03	1.92–2.34	<0.01
**Intraoperative**			
• Length of CPB (per minute)	1.01	1.01–1.02	<0.01
• RBC transfusion	1.41	1.02–1.94	0.03
**pHb-AUC <19**	1.41	1.03–1.93	0.03

AKI: acute kidney injury; BMI: body mass index; CI: confidence interval; CPB: cardiopulmonary bypass; OR: odds ratio; pHb-AUC: perioperative hemoglobin area under the curve; RBC: red blood cells.

Due to the clinical implications of the intraoperative hemoglobin levels to conduct clinical decisions, the importance of its value for pHb-AUC calculation and the identified risk of RBC transfusions, we performed a multivariate linear regression analysis in order to find predictors of minimum intraoperative hemoglobin (Hb2). The results are shown in Table 6. We just found 3 factors independently associated with Hb2: advanced age, diabetes mellitus and preoperative anemia (p <0.05). Unexpectedly, the presence of chronic renal failure, heart failure and the length of CPB were not associated with decreased intraoperative hemoglobin.

**Table 6:** Multivariate linear regression analysis of intraoperative hemoglobin predictors in cardiac surgery adjusted by RBC transfusion.

Variable	B	MSE	p value
Age (years)	- 0.014	0.004	<0.01
Chronic heart failure	- 0.08	0.11	NS
Chronic renal insufficiency	- 0.09	0.15	NS
Diabetes mellitus	- 0.29	0.11	<0.01
Length of CPB (per minute)	- 0.001	0.001	NS
Preoperative Hb (Hb1)	0.29	0.03	<0.01

B: coefficient (3.52; p < 0.01); CPB: cardiopulmonary bypass; Hb: hemoglobin; MSE: Mean Standard Error; NS: non significant; RBC: red blood cells

## Discussion

Our study shows a significant relationship of a pHb-AUC <19 g/dL, with CSA-AKI (OR 1.75, 95% CI: 1.30–2.35, p <0.01) and more significant is the fact that it is the only potentially modifiable hemoglobin determination and independently of RBC transfusion in the multivariate logistic regression analysis, associated with AKI development (OR 1.41 95% CI:1.03–1.93, p = 0.03). The pHb-AUC was also identified as a risk factor for in-hospital mortality and prolonged hospital length of stay.

Previously, Ranucci et al [20], with the aim of assessing the quality of patient blood management in cardiac surgery, described the percentage of haematocrit variation index (PHEVAR). It was built with six haematocrit perioperative values and was assessed as the area under the curve (AUC) of the haematocrit variations. The greater is the PHEVAR index, the larger is the negative haematocrit variation through the perioperative period. They found that the index was an independent predictor of perioperative mortality and significantly higher values of PHEVAR were detected in patients with acute kidney injury. However, the study showed some limitations. In the postoperative transfused patients, they assigned arbitrarily the previous haematocrit for calculation purposes and therefore did not reproduce the real perioperative haematocrit evolution. Additionally, the model excludes the role of preoperative anemia as a risk factor, because they considered the basal haematocrit as the initial value independently of its potential influence in the postoperative outcomes. By contrast, the application of the pHb-AUC concept has several advantages. It is based on real perioperative hemoglobin determinations using a simple formula and it could be used independent of the values for anemia definition. Moreover, the pHb-AUC <19 g/dL was the only hemoglobin measurement independent from RBC transfusion related to CSA-AKI in the multivariate analysis. The AUROC analysis also showed a slightly better performance of the pHb-AUC when is compared to other isolated hemoglobin determinations (Hb1 and Hb2).

The clinical impact of this concept is to recognize that avoiding prolonged anemic periods (“protective AUC”), potentially decreases the risk of AKI development. Accordingly, the pHb-AUC could be used as a surrogate marker of perioperative hemoglobin concentration and might be a better indicator of the “anemic status” than isolated values of hemoglobin.

In clinical practice we could use the minimal perioperative hemoglobin values defined by this theoretical and “protective AUC” to calculate an AUC greater than 19 gr/dl, in order to avoid unnecessary transfusions. To the best of our knowledge, this is the first description of the pHb-AUC in CSA-AKI. Nonetheless, the clinical impact of this “protective AUC” and other potentially modifiable risk factors described in the present study, must be confirmed with larger and well-designed prospective randomized controlled trials.

Pre and intraoperative anemia were also found as risk factors for AKI (OR 2.58 and 1.32, respectively), however, after transfusion adjustment, only the preoperative values remain significant. The relationship between anemia and CSA-AKI are consistent with previous reports. Anemia and RBC transfusion have been identified as important risk factors for CSA-AKI and short-term mortality in this population [9,14]. Recently, a systematic review and meta-analysis based on observational studies [21], described the association between preoperative anemia and postoperative outcomes. In 10 studies including 51787 cardiac surgical patients, preoperative anemia (n = 10197, 19.69%) was associated with an increased risk of death (OR 2.98, 95% CI: 2.02–4.38; I = 84%, p <0.001). Furthermore, in 7 studies including 42931 cardiac surgical patients with AKI as outcome, preoperative anemia (n = 7733, 18.01%) was associated with increased incidence of postoperative AKI (OR 3.75, 95% CI: 2.95–4.76; I = 60%, p < 0.001). These data reinforce findings in the present study. However, some limitations must be considered when interpreting this analysis. The statistical heterogeneity is high, probably due to different definitions of anemia used by included studies. Additionally, the majority are single center and observational studies, it is not possible to account for the potential confounding effect of co-morbid disease, and it remains unclear whether anemia is the cause of poor outcome after CS or a marker of disease severity that increases the risk of AKI.

The prognosis among the patients with CSA-AKI is poor, with a more than 2-fold increase in early and long-term mortality regardless of the AKI definition used [22]. The subgroup of patients who develop AKI requiring RRT have up to a 4-fold increased risk of short-term and long-term mortality [2]. Moreover, previous studies have shown that even mild degrees of kidney dysfunction after CS are associated with increased mortality [23–26]. Therefore, prevention of renal dysfunction is of paramount importance. Several studies have explored the risk factors implicated in the development of CSA-AKI. While, the majority of the risk factors identified are not modifiable [3,10–13], RBC transfusion and anemia are important risk modifiable factors for CSA-AKI and short-term mortality in this population [9,14]. In our Spanish multicenter cohort study, our observations are largely consistent with previous reports.

Karkouti et al. have explored broadly the relationship between preoperative anemia, RBC transfusion and the development of AKI after CS [27–30]. Patients with baseline anemia (Hb <12.5 gr/dL) had 4.1% incidence of AKI as opposed to only 1.6% of non anemic patients. AKI rates increased in direct proportion to the amount of erythrocytes transfused, and this increase was more pronounced in anemic patients [29].

Despite all the information previously described, the interrelationship between these variables and AKI is still confusing, the definition of anemia and the thresholds for transfusion are variable and there are no well-defined protocols for clinical decision-making. Moreover, it remains uncertain whether the treatment of anemia through allogeneic red cell transfusion is associated with harm or benefit and is also unclear whether correcting preoperative anemia can improve outcomes [31, 32].

The results of our multicenter cohort study describe potentially modifiable risk factors in terms of anemia and hemoglobin evolution in CSA-AKI. The multivariate regression logistic analysis showed the influence of preoperative hemoglobin in the development of significant IOA. Therefore, it is possible that preoperative optimization regarding the diagnosis and treatment of anemia could have a positive impact in postoperative evolution.

However, our study has several limitations to be considered. Neither the cause nor the duration of preoperative anemia, each of which may have prognostic implications, was known. Our results, accordingly with a retrospective analysis, could have potential bias due to the absence of data regarding the influence of possible confounding variables that affect kidney function, such as: hemodynamic variations during CPB, the priming volume, vasopressor doses, perioperative fluid balance or differences in the dose of colloids. As an observational study, we did not use a specified protocol to guide transfusion therapy neither priming volume across all participating sites; therefore, we cannot determine which patients underwent transfusion due to blood loss or CPB-induced hemodilution associated with the priming volume. Finally, the results should not be generalized to other populations because only patients undergoing CS with CPB were included.

We can conclude that the perioperative hemoglobin area under the curve is an independent predictor of AKI, mortality and prolonged length of hospital stay in cardiac surgery.
